# Light-responsive sodium butyrate-loaded manganese porphyrinic HOF for enhanced antibacterial and biofilm-inhibitory activity

**DOI:** 10.3389/fchem.2026.1941361

**Published:** 2026-08-20

**Authors:** Sishi Liu, Yu Yao, Hongliang Shen, Xin Zhang

**Affiliations:** 1 Department of Pathology, The First Affiliated Hospital of Harbin Medical University, Harbin, China; 2 Department of Orthopedics, The Second Affiliated Hospital of Harbin Medical University, Harbin, China; 3 College of Chemistry, Chemical Engineering and Resource Utilization of Northeast Forestry University, Harbin, China; 4 Department of Orthopedics, The First Affiliated Hospital of Harbin Medical University, Harbin, China

**Keywords:** bacterial viability, biofilm inhibition, hemolytic activity, hydrogen-bonded organic framework, light-responsive antibacterial material, Mn-TCPP, sodium butyrate

## Abstract

Bacterial infections of biofilms are hard to treat as the matrix and physiological differences in the cells within a biofilm limit the ability of traditional antibiotics to control the growth of bacteria. We synthesized a sodium butyrate-encapsulated manganese tetrakis (4-carboxyphenyl) porphyrin hydrogen-bonded organic framework (NaB@Mn-TCPP HOF), which was used to determine its physical and chemical properties, guest-loading capacity, release behavior, hemolytic effects, antibacterial activity and anti-biofilm formation of *Escherichia coli* and *Staphylococcus aureus*. The NaB incorporation was confirmed by Fourier-transform infrared spectroscopy, powder X-ray diffraction, ultraviolet-visible spectroscopy, dynamic light scattering, zeta-potential measurement, elemental mapping, and other methods demonstrated that the parent Mn-TCPP HOF retained most of its principal spectroscopic, solid-state diffraction, and colloidal features. Indirect spectrophotometry of absorbance of UV light revealed an apparent loading of NaB of 56 percent with respect to the mass of carrier at feed ratio of 4 mg NaB per 5 mg carrier corresponding to an estimated NaB content of about 36 wt% in the recovered composite and an apparent encapsulation rate of about 70%. About half of the calculated loaded NaB was released gradually over 12 h without reaching the final plateau. In a five-group design that is exploratory and not dose-matched, images of the colonies were taken after exposure to 660 nm irradiation at 100 mW cm^−2,^ and the results indicated the reduction of colony development, crystal-violet staining, propidium-iodide-associated signal, and surface-related bacterial coverage in the NaB@Mn-TCPP HOF group. Biofilm experiment was performed on the inhibition of a new biofilm in the course of 24 h instead of destroying an already matured one. After 2 hours of exposure, less than 5 percent hemolysis was produced by the composite at 100 micrograms mL ^−1^. These findings can be used to make further studies of NaB@Mn-TCPP HOF as a combined material platform that reacts to light. It has not been clarified by the current design whether reactive oxygen species, manganese excretion, the pathway of bacteria controlled by NaB, or pharmacological interactions were involved.

## 1 Introduction

The antimicrobial resistance is a significant problem in the world. The systematic review predicted that there was about 1.27 million deaths and 4.95 million deaths related to bacterial antimicrobial resistance in the year 2019 (Antimicrobial Resistan ce, 2022). It becomes even more difficult to treat when the bacteria live as biofilm. These cells are adherent on surfaces, where they reside within a matrix of polysaccharides, proteins, lipids, extracellular nucleic acids, which can be used to adhere to each other, transfer nutrients, communicate with one another and resist stressors (Flemming et al., 2016). Biofilm tolerance has only limited penetration of antimicrobials. Other factors such as oxygen and nutrient gradients also lead to the presence of metabolically heterogeneous subpopulations (slow-growing and persister-like) that do not respond well to treatment targeting active biosynthetic pathways (Flemming et al., 2016; Stewart and Costerton, 2001). Such characteristics have been the reason behind the use of non-antibiotic methods, which involve fast physical or chemical damage, and inhibition of the formation of biofilms.

Antimicrobial photodynamic therapy is a way to go. The photosensitizer, after being excited by light, will be in the excited state and can release energy or electrons to the surrounding substrates and oxygen molecule, producing reactive oxygen species (ROS) like singlet oxygen, superoxide, hydrogen peroxide, hydroxyl radicals (Sabino et al., 2023; Wainwright et al., 2017). These oxidants have a short life time and can reach the membrane of bacteria as well as proteins, nucleic acids, and other redox active metabolites in the zone that is exposed to irradiation. Photodynamic damage is not necessarily targeted on one cell type as compared to antibiotics which are more specific and thus it could be less susceptible to single molecular target; however, sublethal doses and microbial adaptation to stress should be taken into account (Kashef and Hamblin, 2017; Maisch, 2015). Porphyrins are good photosensitizers since they possess conjugated macrocycles, absorb visible light and have chemically accessible peripheral groups. Tetrakis (4-carboxyphenyl) porphyrin (TCPP) also has four carboxyphenyl units that allow supramolecular formation. A particulate structure can help to retain the particles and prevent them from aggregating freely and provide the opportunity to introduce an agent that would modulate biofilm (Beirao et al., 2014; Pereira et al., 2014).

A possible architecture of organizing these elements is the hydrogen-bonded organic framework (HOFs). HOFs are unlike covalent organic frameworks that have been constructed by covalent bonding or metal-organic frameworks that rely on a coordination between metal and ligand. Their ability to crystallize under gentle conditions as well as post-synthetic guest inclusion can be allowed by their noncovalent interactions, which are reversible. The porphyrins with peripheral carboxylic-acid groups are also very suitable in this approach since the carboxyl groups would interact through hydrogen-bonding structures and the planar aromatic cores would provide further stacking effects and high concentration of photoactive chromophores (Lin et al., 2022; He et al., 2019). Other studies in eCM have reported the delivery of a near-infrared activated metal-organic-framework nanoagent via microneedles for anti-biofilm infection treatment, showing how the porous carrier and the external stimulation of antimicrobial action are being used more generally (Chen et al., 2026). Alongside it, the latest work of eCM concerning porous iron scaffolds with manganese and copper has combined physicochemical analysis with biocompatibility testing, where the authors underline the importance of studying the material and biological properties of the metal-containing biomaterials (Grodzicka and G. GąsiorT. JędrzejewskiS. WrotekP. MadajskiA. Radtke, 2025). There are other types of HOFs that were made from Mn-TCPP as a biologically active supramolecular system (Ya et al., 2024).

Manganese has functional potential as well as mechanistic uncertainty. Manganese porphyrins have the ability to exhibit redox, catalytic, imaging and stimulus-responsive properties (Xu et al., 2021; Ya et al., 2024), however it is not possible to conclude that manganese exposure alone would be bactericidal without information on chemical form and concentration. Pathogenic bacteria actively control manganese intake due to the fact that manganese facilitates metalloenzyme activity and oxidative-stress tolerance (Juttukonda and Skaar, 2015; Turner et al., 2015). On the other hand, antibacterial action of manganese dioxide nanostructures can be attributed to surface reactions, membrane interactions, catalytic ROS production, or oxidative dissolution (Du et al., 2020), which cannot be directly extrapolated to manganese coordinated in a porphyrin HOF. Metalation can also change porphyrin excited-state behavior and change the quantity or type of photochemical products. The current study therefore considers ROS identity, manganese release, and manganese-mediated biological effects as unresolved mechanistic issues rather than established processes.

NaB has a chemically separate aspect, which is said to be an antimicrobial and biofilm inhibitory, as well as antivirulence effect on the basis of the microbial species, concentration, medium composition, and pH. In *Vibrio* parahaemolyticus, NaB has been reported to decrease motility, extracellular-polysaccharide, surface hydrophobicity, quorum-sensing activity, and gene expression in relation to biofilms (Zhu et al., 2022). The inhibition of biofilm development by *Salmonella Typhimurium* has also been observed in the lab and food model (Liu et al., 2022) as well as the presence of antimicrobial action in pathogenic yeasts (Nguyen et al., 2011). ToxT-dependent virulence processes are not necessarily inhibited by NaB in *Vibrio cholerae* (Kundu et al., 2025). Such findings would justify the likelihood that NaB might have an impact on the phenotypes or biofilm growth of bacteria in addition to any direct antibacterial actions. It may be introduced into a HOF, but the location of the guest loaded must be considered with caution and its state at the molecular level.

The combination of NaB and an Mn-TCPP HOF can thus be a combination of a light-sensitive porphyrinic component with a small molecule that is related to lower biofilm formation. Nevertheless, better performance of a composite as compared to the treatment groups does not necessarily mean that there is pharmacological synergy. To demonstrate synergy, dose-matched combination matrices and a quantitative reference model (Bliss independence, Loewe additivity, or combination-index approach) are required (Chou, 2010; Foucquier and Guedj, 2015). Without such analyses, the best possible description would be an improved or collaborative phenotype in the conditions tested.

**FIGURE 1:**
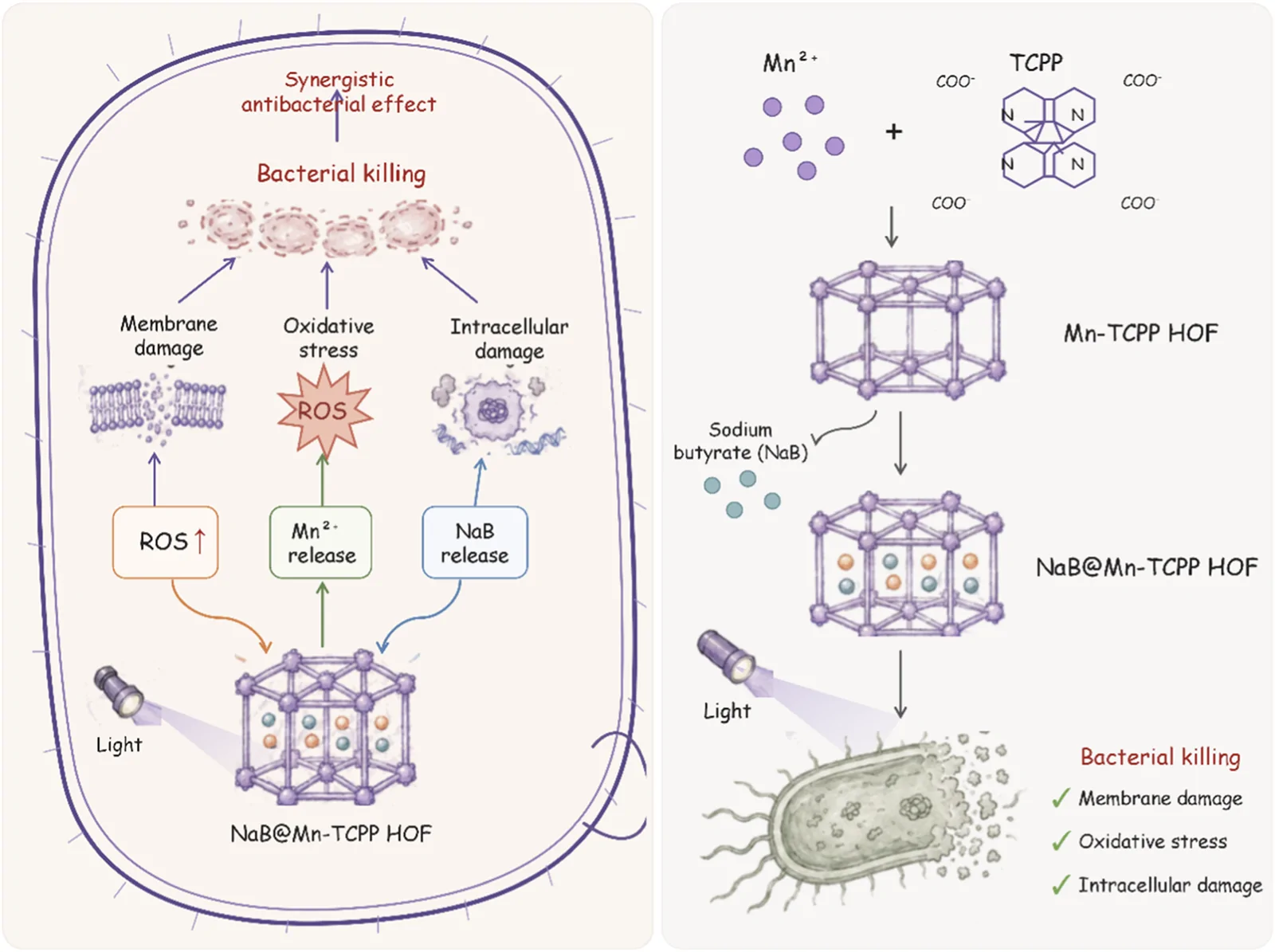
A diagram of the process of building and its biological interpretation in the case of NaB@Mn-TCPP HOF. The light-associated, ROS-based, manganese-based and NaB-related processes are not measured directly but are hypotheses. This is a conceptual model only.

This was a proof-of-concept experiment in which we synthesized NaB@Mn-TCPP HOF and characterized its spectroscopic, diffraction, colloidal, elemental properties, the loading and release of NaB, hemolytic activity and antibacterial, biofilm-inhibitory phenotypes were analyzed to *Escherichia coli* ATCC 25922 and *Staphylococcus aureus* ATCC 25923. The comparison of the biological effects consisted of the untreated group, light-only group, free-NaB group, Mn-TCPP HOF illuminated, and NaB@Mn-TCPP HOF illuminated. This is an exploratory design that did not factorially but rather described the material-related phenotypes observed under one or another set of conditions and it did not isolate the dark toxicity, light-specific action, assembly-specific action, component interaction. It was not possible to measure specific species of ROS, manganese liberation, signaling pathways of NaB, eradication of mature-biofilms, and quantitative synergy (Figure 1).

## 2 Materials and methods

### 2.1 Materials, reagents, and bacterial strains

The Mn-TCPP was ordered in MedChemExpress (purity 98%; catalog no. HY-W075699). The NaB was bought at Sigma-Aldrich (St. Louis, MO, United States; purity of the product is 98.5 percent; catalog no. B5887). Organic solvents and buffer salts and other reagents were prepared in analytical grade. Calcein - AM/PI bacterial viability staining kit, crystal violet, phosphate - buffered saline (PBS), and normal saline (NS) were procured by Beijing Jumei Biotechnology Co., Ltd (catalog no. MF939-01), Sigma - Aldrich (catalog no. C0775), Gibco (catalog no. 10010023), and Oxoid (catalog no. EB0334G). TCPP used as control group was bought in Tokyo Chemical Industry Co., Ltd (purity more than 97%; catalogue number A5015). The experiments were done with ultrapure water (UPW).

American Type Culture Collection (ATCC, Manassas, VA, United States) had *E. coli* ATCC 25922 and *S. aureus* ATCC 25923. They were both grown in Luria-Bertani broth or on Luria-Bertani agar (BD Difco; catalog nos. 244620 and 244520 respectively). The hemolysis test was done with the help of Thermo Fisher Scientific commercial sterile defibrinated sheep whole blood which is cataloged under the number R54008. It did not include any human subject or human samples. Institutional biosafety and ethical standards were followed in the use of biological material derived from animals.

### 2.2 Preparation of Mn-TCPP HOF

Mn-TCPP (20 mg) was put in or dissolved in 10 mL of N,N-dimethylformamide (DMF) at the temperature of 25 °C and bath sonicated at low power for 20 min. The preparation was then poured into 40 mL UPW, which is an antisolvent, with a rate of 1 mL per minute at 600 rpm and mixed under stirring. It was pH adjusted to 3.0 by HCl concentration of 0.1 mol L ^−1^ to induce self-assembling based on hydrogen bonding and other noncovalent interactions and kept in it overnight.

The product was centrifuged at 12,000 × g and 4 °C during 10 min, washed thrice with UPW, and freeze-dried at −50 °C and less than 20 Pa. The Mn-TCPP HOF that was recovered was kept in dry conditions and under light.

### 2.3 Preparation of NaB@Mn-TCPP HOF

Mn-TCPP HOF (5 mg) was dispersed in 10 mL UPW to give a carrier concentration of 0.5 mg mL^−1^. NaB was added in feed amounts of 0, 2, 4, 6, 8 or 10 mg. Mixtures were incubated at 25 °C and 150 rpm for 12 h in the dark.

After incubation, the samples were centrifuged at 12,000 g. The precipitate was washed twice with UPW to remove loosely associated NaB and freeze dried to obtain NaB@Mn-TCPP HOF. The supernatant and wash fractions were combined for indirect quantification of unbound NaB.

### 2.4 Estimation of apparent NaB loadin

NaB standard solutions were prepared in the relevant solvent matrix, and absorbance was measured at 210 nm to generate a calibration curve. The supernatant and wash fractions obtained after each loading experiment were combined and analyzed using the same procedure to estimate the mass of unbound NaB. Since this indirect ultraviolet method is not chemically specific for butyrate in a complex matrix, the resulting values are reported as apparent estimates.

The mass of seemingly loaded NaB (m _loaded_) was determined as m _loaded_ = m _0_ – m _f_. The apparent loading over carrier mass was determined in the following manner:

$$\text{Apparent carrier}-\text{mass}-\text{based loading }\left(\%\right)=\left(m_{\text{loaded}}\;/\;m_{c}\right)100\%$$

where m0 is the initial NaB mass, mf is the estimated mass of unbound NaB in the combined supernatant and wash fractions, and mc is the Mn-TCPP HOF carrier mass. The calculated NaB content in the recovered composite was [m _loaded_ / (m _c_ + m _loaded_)] 100%, and the apparent encapsulation efficiency was [m _loaded_ / m _0_] 100%. Three parallel samples were used to analyze each feed condition, and results are given as mean standard deviation (SD).

### 2.5 Fourier-transform infrared spectroscopy

The samples of fully dried Mn-TCPP HOF and NaB@Mn-TCPP HOF were ground with spectroscopic-grade KBr and pressed into pellets. The Fourier-transform infrared (FTIR) spectra were obtained in the range of 5004, 000 cm^−1^ at a resolution of 4 cm^−1^ with an accumulation of 32 scans using a Nicolet iS50 spectrometer (Thermo Fisher Scientific, United States). A background of KBr was measured prior to measurement of the sample and all spectra underwent the same baseline-correction and normalization.

### 2.6 X-ray diffraction

Dried samples were powdered and loaded onto the sample holder. Powder XRD patterns of Mn-TCPP HOF and NaB@Mn-TCPP HOF were measured on a Bruker D8 ADVANCE diffractometer (Bruker, Germany) equipped with a Cu K radiation source operating at 40 kV/40 mA. Data were acquired from 5 to 90 in 2 with a step size of 0.02 and a scanning speed of 8 min. The same acquisition and processing parameters were applied to both samples.

### 2.7 Ultravioletvisible absorption spectroscopy

Mn-TCPP HOF and NaB@Mn-TCPP HOF were dispersed in deionized water at 20 μg mL^−1^ and sonicated in a low-power bath sonicator for 10 min. Samples were transferred to 1 cm quartz cuvettes and UVvis absorption spectra were collected from 200 to 850 nm on a U-4100 spectrophotometer (Hitachi, Tokyo, Japan) using deionized water as the blank. Sample concentration, optical path length, scan conditions, and baseline correction were held constant between groups.

### 2.8 Hydrodynamic particle size measurement

The hydrodynamic particle size was determined using dynamic light scattering based on the general principles of ISO 22412:2025. Mn-TCPP HOF and NaB@Mn-TCPP HOF were suspended in deionized water at a concentration of 0.1 mg mL ^−1^, sonicated in low-power bath at 10 min, and equilibrated at 25 °C at 2 min. The measurements were done with Litesizer 500 instrument (Anton Paar, Graz, Austria). Each sample had three independent measurements that included 10 submeasurement cycles. Findings are given as mean SD.

### 2.9 Zeta-potential measurement

The Mn-TCPP HOF and NaB@Mn-TCPP HOF were dispersed in the same medium used for particle size measurements, keeping sample concentration, pH and ionic strength constant. Zeta potential was measured by electrophoretic light scattering on a Litesizer 500 instrument equipped with disposable folded capillary cells at 25 °C. Each sample was measured three times independently and results are reported as mean s.d.

### 2.10 HAADF-STEM imaging and EDS elemental mapping

NaB@Mn-TCPP HOF was dispersed in anhydrous ethanol by low power sonication for 10 min. An aliquot of the dispersion was placed on a carbon-coated copper grid and dried at room temperature. Morphology was investigated by high-angle annular dark-field scanning transmission electron microscopy (HAADF-STEM) with a Talos F200X G2 microscope (Thermo Fisher Scientific, United States) operated at 200 kV.

Elemental mapping was carried out with the respective EDS system. The spatial distribution of C, O, N, Mn and Na was registered from the same area as HAADF-STEM image.

### 2.11 *In vitro* release of NaB

NaB@Mn-TCPP HOF having a known estimated quantity of NaB was put into a dialysis membrane that had a molecular-weight cutoff of 3.5 kDa and immersed in PBS (pH 7.4). The overall external release-medium volume was 20 mL. The samples were incubated at 37 °C with shaking at 100 rpm, and the aliquots of the external medium were taken at 0, 2, 4, 6, 8, 10, and 12 h.

At every sampling point, the external medium that was removed was replaced by an equal volume of fresh PBS pre-equilibrated at 37 °C. The ultraviolet absorbance technique outlined in loading analysis was used to estimate released NaB. The repeated sampling and replacement of the medium were corrected on cumulative released mass.

The cumulative release percentage was calculated as follows:

$$\text{Cumulative release }\left(\%\right)=\left(\text{Mt }/\text{ ML}\right)\;100\%$$

where Mt is the cumulative estimated mass of NaB released at time t and ML is the initial estimated mass of NaB loaded in the material. Three parallel samples were analyzed, and results are presented as mean SD.

### 2.12 Hemolysis assay

The test of hemolysis was carried out based on the standards provided by ASTM F756-17 (2025) and other tests to determine hemolysis caused by nanoparticles. Plasma was removed by centrifuging whole blood at 4 °C at 1,500 × g for a period of 10 minutes. The erythrocytes were also washed three times with NS to make it clear and then diluted to get an erythrocyte suspension of 2% (v/v).

The positive control of complete-hemolysis was UPW and the negative control was NS. The experimental groups were TCPP, NaB (free), Mn-TCPP HOF and NaB@Mn-TCPP HOF at 100 g mL ^−1^.

The suspension of erythrocytes was combined with the treatment in a ratio of 1:1 and left to incubate at 37 °C for 2 hours. The centrifuge was used to spin the samples at 1,500 x g during 10 min, then the supernatants were photographed and the absorbance was taken at 540 nm.

The hemolysis percentage was calculated as follows:

$$\text{Hemolysis }\left(\%\right)=\left[\left(A_{\text{sample}}–\;A_{\text{negative}}\right)/\;\left(A_{\text{positive}}–\;A_{\text{negative}}\right)\right]100\%$$

The three parallel samples were analyzed in every group and the results are represented as mean SD. The interpretation was limited to tested concentration and exposure conditions.

### 2.13 Bacterial culture and experimental groups

*E. coli* and *S. aureus* were grown in LB broth separately at 37 °C shaking at 180 rpm for about 12 h. The overnight cultures were diluted to a ratio of 1:100 in fresh LB broth and incubated further for 24–48 h until the logarithmic growth phase is achieved. Centrifugation at 5,000 x g was used to collect cells after which they were washed with sterile PBS and resuspended. OD600 readings and plate counting were employed to adjust the starting suspension that was used in the planktonic and biofilm-formation assays to approximately 1 × 106 CFU mL ^−1^.

There were five experimental groups that were tested, including: (1) Control, no material and no irradiation; (2) Light, irradiated without material; (3) NaB, free NaB treatment; (4) Mn-TCPP HOF, Mn-TCPP HOF treatment and irradiation; and (5) NaB@Mn-TCPP HOF, composite and irradiation. The end concentrations were 0.5 mg mL ^−1^ of free NaB and 100 µg mL ^−1^ of Mn-TCPP HOF and NaB@Mn-TCPP HOF. According to the calculated NaB content of about 36 wt% in the recovered composite, the NaB@Mn-TCPP HOF treatment would have provided an approximate NaB-equivalent concentration of about 36 µg mL ^−1^. The free-NaB and composite groups were hence not dose matched on NaB. The comparison of five groups was not a full factorial design of materials by illumination, and all biological comparisons were viewed as exploratory.

Following 30 min of preincubation with the suspensions of bacteria, Light, Mn-TCPP HOF, and NaB@Mn-TCPP HOF groups were exposed to a source-to-sample distance of 10 cm, a wavelength of 660 nm and an irradiance of 100 mW cm^−2^ at the same time. The Control and NaB groups were also kept in darkness under similar conditions in the laboratory during the same period. There was no quantitative measure of irradiance uniformity, spectral bandwidth and sample temperature and thus there was no attempt to deduce a therapeutic window or eliminate a thermal effect.

### 2.14 Colony-forming unit assay

Bacterial suspensions were serially diluted tenfold following treatment. Aliquots of 100 µL of suitable dilutions were spread on LB agar and incubated at 37 °C over 24 h. Representative plates were photographed. Full per-replicate colony-count records, plating limits of detection, rules of plates beyond the countable range, and zero-colony handling were not available in the current report; therefore, the retained plates were interpreted qualitatively and no numerical bactericidal endpoint was determined.

The antibacterial rate was calculated as follows:

$$\text{Antibacterial rate }\left(\%\right)=\left[\left(N_{\text{control}}\;-\;N_{\text{sample}}\right)/\;N_{\text{control}}\right]\;100\%$$

where N control and N sample are the viable counts of bacteria in the control and sample respectively. The corrected equation is given as a methodological correction, the bactericidal percentage and log10 reduction are not indicated due to the fact that quantitative CFU data was not available.

### 2.15 Calcein-AM/PI viability staining

The bacterial cells were harvested by centrifugation at 5,000 x g and then washed twice in sterile PBS. The working solutions of calcein-AM and propidium iodide (PI) were made as per the guidelines provided by the manufacturer. Cells were stained in darkness over a period of 30 min, rinsed and placed on glass slides or imaging dishes.

The confocal laser-scanning microscope of Leica TCS SP8 (Leica Microsystems, Germany) was used to capture the images. Calcein-AM fluorescence served as an indicator of the integrity of membranes that are not very broken whereas PI fluorescence showed a breakage in membrane integrity. The time of exposure, gain on detector, power of laser and processing of images were fixed among the groups. The pictures that remained were judged qualitatively; no inferential data about the amount of fluorescence, single-stain dataset, or material-only fluorescence control is presented.

### 2.16 Crystal violet biofilm assay

The same suspension of bacteria as above was then inoculated into the 96-well plates and treated as per the experimental groups. The irradiation at 660 nm was performed in the biofilm-formation test, after the material-bacterium preincubation that lasted 30-min and prior to the incubation of the static 24 h at 37 °C. It was then washed with PBS three times to remove nonadherent cells by removing the supernatant. The retained record of the experiment did not quantify evaporation and plate-edge effects.

Biofilms that were developed were fixed using the methanol at 15 min and crystal violet was stained with 0.1 percent (w/v) in 15 min. The plates were washed off of excess dye, then they were air-dried and taken photographs. The plate pictures were kept only to compare the attached biomass qualitatively during the formation of biofilms. There is no inferential OD570, blank-well adjustment or viable-biofilm estimate.

### 2.17 Scanning electron microscopy

The cells of the bacteria or the biofilms in development were harvested by using sterile silicon wafers at 4 °C with glutaraldehyde (2.5%) overnight, after which they were washed in PBS and then dehydrated in a series of 30%, 50%, 70%, 80%, 90% and 100% ethanol.

The samples were sputter-coated with gold and viewed under a field-emission scanning electron microscope (SU8010, Hitachi High-Technologies, Japan) after critical-point drying. The accelerating voltage, working distance, magnification and acquisition settings were as similar as possible among the treatment groups. The images were analyzed qualitatively.

### 2.18 Statistical analysis

Quantitative data on material-characterization, release and hemolysis are given as mean 3 SD of three independent measurements or parallel samples, as described in each assay. The image-based colony, viability-staining, crystal-violet, and scanning electron microscopy observations did not have complete numerical datasets to be used; these results were thus explained qualitatively and were not subjected to inferential statistical testing. Different image fields of one specimen were not considered as independent biological replicates.

GraphPad Prism version 9.0 was used for data plotting and descriptive analysis.

## 3 Results

### 3.1 FTIR analysis

**FIGURE 2:**
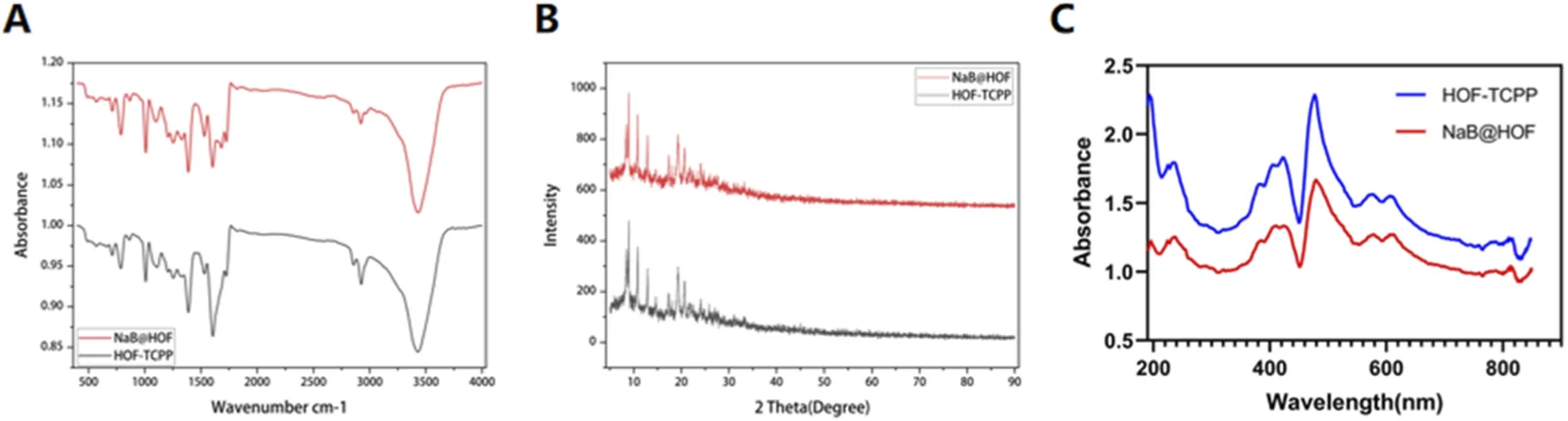
Mn-TCPP HOF and NaB@Mn-TCPP HOF Physicochemical analysis. **(A)** Fourier transform infrared spectrums. **(B)** X ray diffraction patterns of powder. **(C)** Ultraviolet-visible absorption spectrum.

As indicated in Figure 2A, Mn-TCPP HOF and NaB@Mn-TCPP HOF displayed largely comparable FTIR profiles at 500–4000 cm^−1^ which means that the main chemical characteristics were preserved following the incorporation of NaB. The relative intensity and local shape of a few bands changed indicating alteration of the local chemical environment or noncovalent interactions. The spectra are not able to distinguish between the specific binding site of NaB or indicate the creation of new covalent bonds.

### 3.2 XRD analysis

As illustrated in Figure 2B, NaB@Mn-TCPP HOF maintained the general diffraction pattern of Mn-TCPP HOF without any significant new sharp peaks. This finding shows that NaB incorporation did not result in an apparent large-scale modification of the parent solid-state organization. The data are compatible with the presence of NaB as a dispersed or poorly crystalline material in internal, interlayer, or surface-associated regions. Since the patterns are vertically offset, peak intensities cannot be compared quantitatively.

### 3.3 UVVisible spectroscopic analysis

Both Mn-TCPP HOF and NaB@Mn-TCPP HOF retained the main porphyrinic absorption characteristics in the range of 200–850 nm (Figure 2C). The loading of NaB was accompanied by variations in total absorbance and shape of peaks, which can be attributed to differences in effective concentration, particle dispersion, light scattering or local environment of the porphyrin units. These spectra show that optical absorption is preserved but they do not prove generation, identity, or amount of ROS.

### 3.4 Hydrodynamic particle size

**FIGURE 3:**
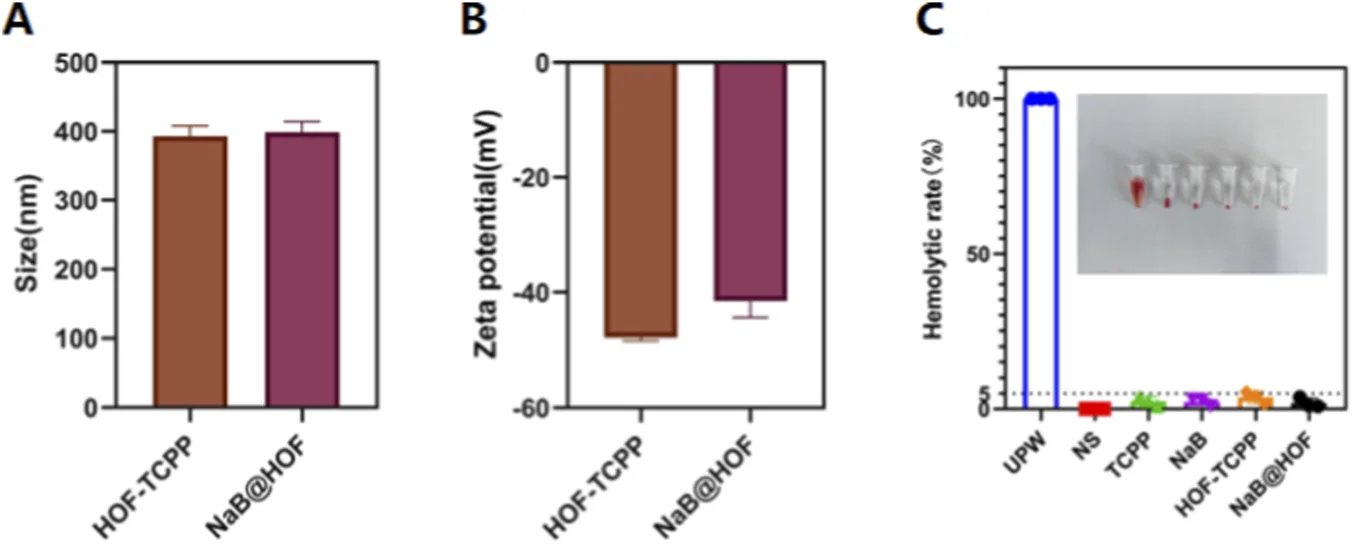
Hydrodynamic size, zeta potential and hemolysis measurements of Mn-TCPP HOF and NaB@Mn-TCPP HOF. **(A)** Average hydrodynamic diameter as measured by dynamic light scattering. **(B)** Zeta potential. **(C)** Hemolysis values and representative supernatants of ultrapure water (UPW), normal saline (NS), TCPP, NaB, Mn-TCPP HOF, and NaB@Mn-TCPP HOF. The dashed line is 5% hemolysis. Data are presented as mean SD (n = 3).

The average hydrodynamic sizes of Mn-TCPP HOF and NaB@Mn-TCPP HOF were about 390 and 395 nm, respectively (Figure 3A; n = 3). The overlapping error bars show that the loading of NaB was not accompanied by a significant change in the size of hydrodynamics under the tested aqueous conditions. Dynamic light scattering is an indicator of suspended-particle behavior and should not be directly related to electron microscopy dimensions.

### 3.5 Zeta potential

The average zeta potentials of Mn-TCPP HOF and NaB@Mn-TCPP HOF were about −48 and −41 mV respectively (Figure 3B; n = 3). The composite was negatively charged after loading, but the negative potential was less in magnitude. This change is in line with alteration of the particle water interface upon incorporation of NaB.

### 3.6 Morphology and elemental distribution

**FIGURE 4:**
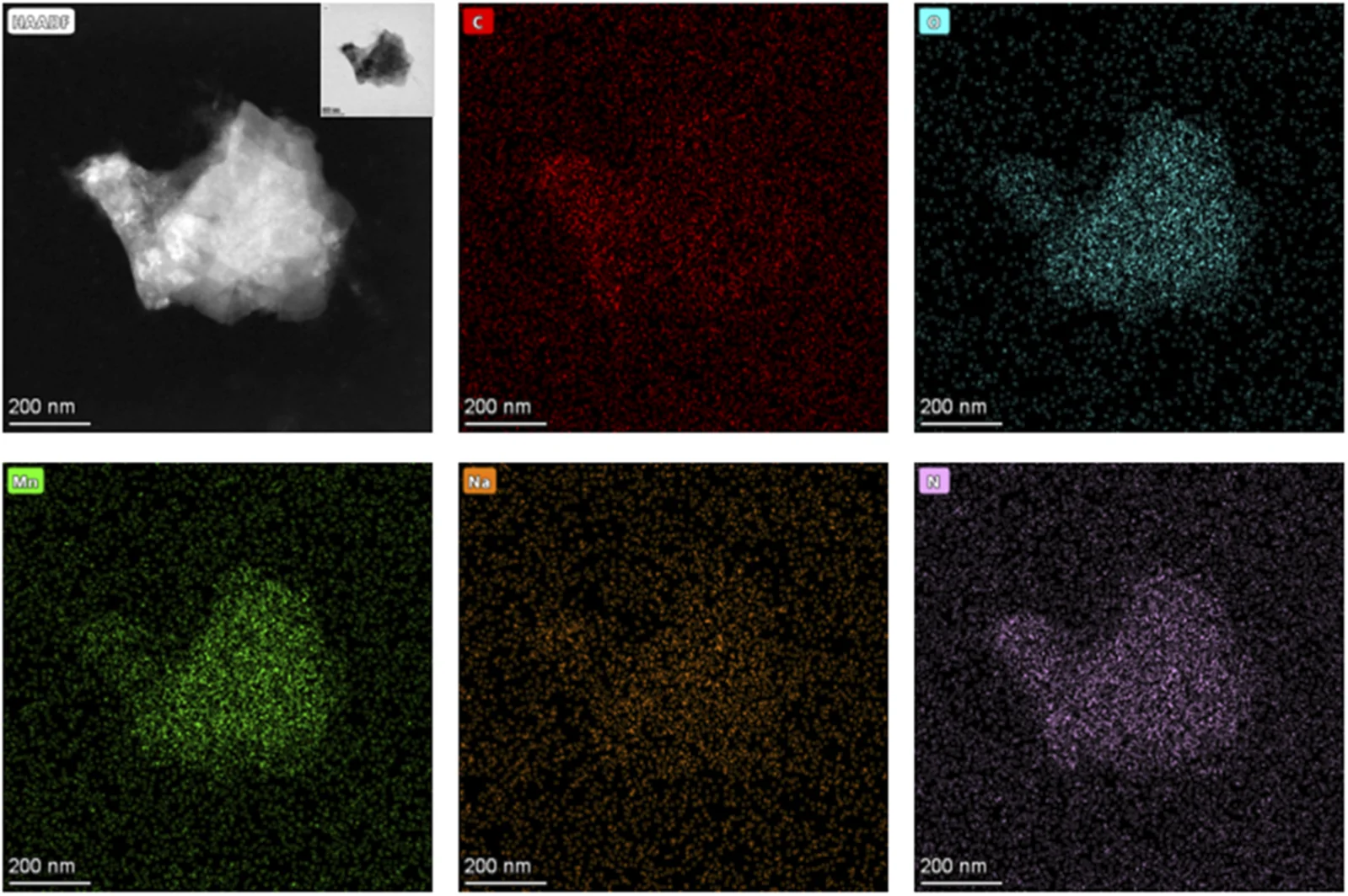
NaB@Mn-TCPP HOF morphology and elemental distribution. HAADF-STEM image and EDS elemental maps of C, O, Mn, Na, and N obtained in the same area of a particle. Scale bars, 200 nm.

HAADF-STEM revealed NaB@Mn-TCPP HOF to be irregular sheet-like or aggregated particles of several hundreds of nm local dimension (Figure 4). EDS mapping revealed spatial overlap of C, O, N and Mn signals with the particle areas, along with a Na signal associated with the same areas.

The elemental distribution, which is combined with the indirect loading estimate and spectroscopic results, favors the association of NaB with the Mn-TCPP HOF particles. Nevertheless, the signal of Na by itself does not determine the chemical state or exact position of butyrate, nor it can differentiate internal loading from interlayer or surface association.

### 3.7 NaB loading

**FIGURE 5:**
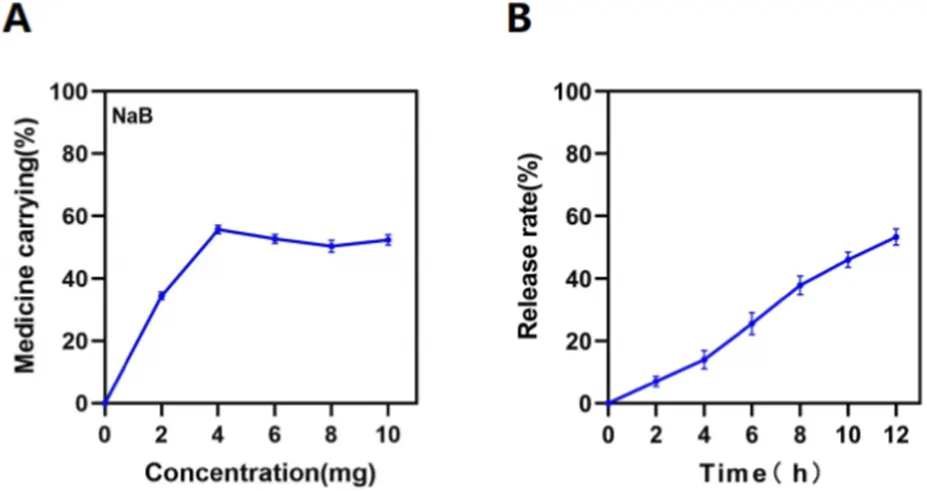
NaB loading and *in vitro* release profile of NaB@Mn-TCPP HOF as observed. **(A)** Apparent carrier-mass-based NaB loading at various initial NaB feed amounts. **(B)** Total estimated NaB release of NaB@Mn-TCPP HOF after 12 h. The data are given as mean SD (n = 3).

Figure 5A indicates that apparent carrier-mass-based NaB loading was dependent on feed amount and then reached a plateau. The values of apparent loading were about 35, 56, 53, 50 and 52 at the NaB feed amounts of 2, 4, 6, 8 and 10 mg respectively. When the feed is 4 mg with 5 mg carrier, the apparent loading of around 56% is equivalent to an estimated loaded mass of NaB of 2.8 mg, an estimated content of NaB of about 36 wt% in the recovered composite, and an apparent encapsulation efficiency of about 70%.

The saturation of the accessible association or confinement sites at a higher feed amount is also reflected by no further increase in this case. Since loading was not measured directly but estimated through ultraviolet absorbance at 210 nm, the figures are expressed as apparent and not as absolute loading capacities.

### 3.8 NaB release

NaB@Mn-TCPP HOF exhibited progressive partial release within 12 h (Figure 5B). The cumulative estimated values of the releases at 2, 4, 6, 8, 10 and 12 h were about 7, 14, 26, 37, 45 and 53 percent respectively (n = 3).

The graph was not found to be near complete instant release and it was still growing in the course of the observation. Since the experiment had been conducted up to 12 h, but there was no terminal plateau or full mass recovery, the data is indicative of progressive partial release as opposed to a definite sustained or controlled release and no particular kinetic model could be identified.

### 3.9 Hemolysis

Figure 3C indicates that the hemolysis was almost complete, with the mean values of hemolysis in NS, TCPP, NaB, Mn-TCPP HOF and NaB@Mn-TCPP HOF being less than the reference value of 5% under the conditions tested (n = 3). The supernatants of material-treatment were not as red as the positive control of UPW.

The data shows that the erythrocytes are low at 100 μg/mL concentration and after a period of 2 hours. This conclusion is only applicable to the tested concentrations, time in incubation and conditions of the test and cannot be generalized to biocompatibility or safety.

### 3.10 Antibacterial activity

**FIGURE 6:**
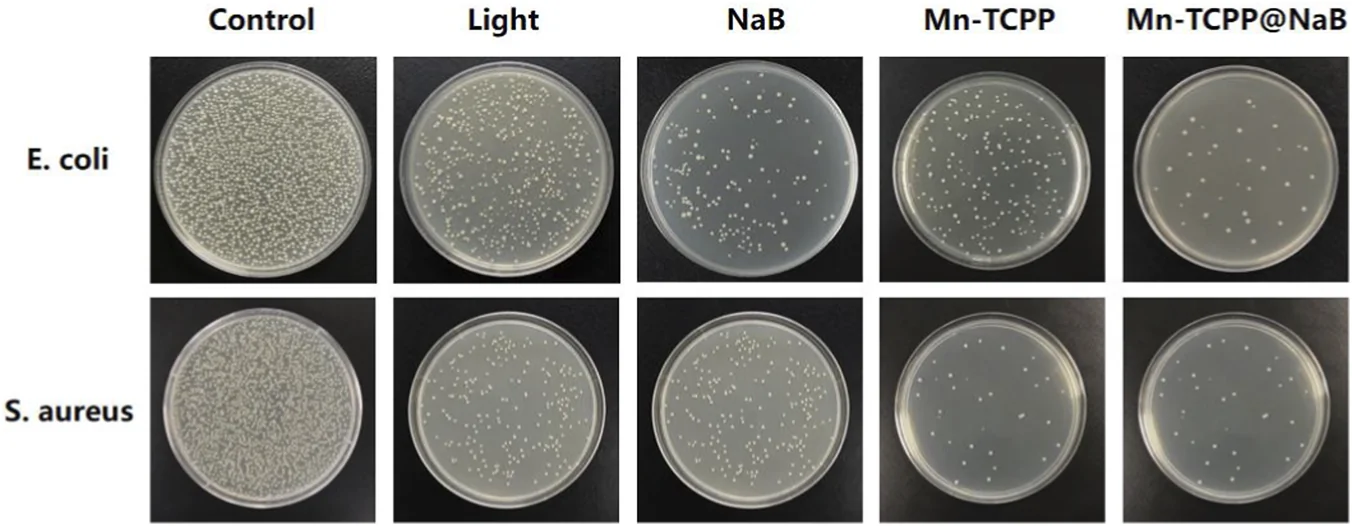
Colony growth of *Escherichia coli* and *Staphylococcus aureus* following the treatments indicated. Columns represent Control, Light, free NaB, Mn-TCPP HOF and NaB@Mn-TCPP HOF and rows are represented by two species of bacteria. Bacterial suspensions were diluted serially and seeded on LB agar and incubated at 37 °C for a period of 24 h after treatment. The images can be read as qualitative.

Representative colony formation images are shown in Figure 6. Both *E. coli* and *S. aureus* Control and Light groups maintained abundant visible colonies. Free-NaB and illuminated Mn-TCPP HOF groups exhibited a clear decrease in colony formation and fewer visible colonies appear in the shown fields on the illuminated NaB@Mn-TCPP HOF plates.

Since full per-replicate CFU data and statistics were not available, the plates are viewed as qualitative observations under the chosen, non-dose-matched and non-factorial conditions. No bactericidal percentages, log10 reductions, loading superiority, light-specific effects or synergy are inferred from the representative images.

### 3.11 Bacterial viability staining

**FIGURE 7:**
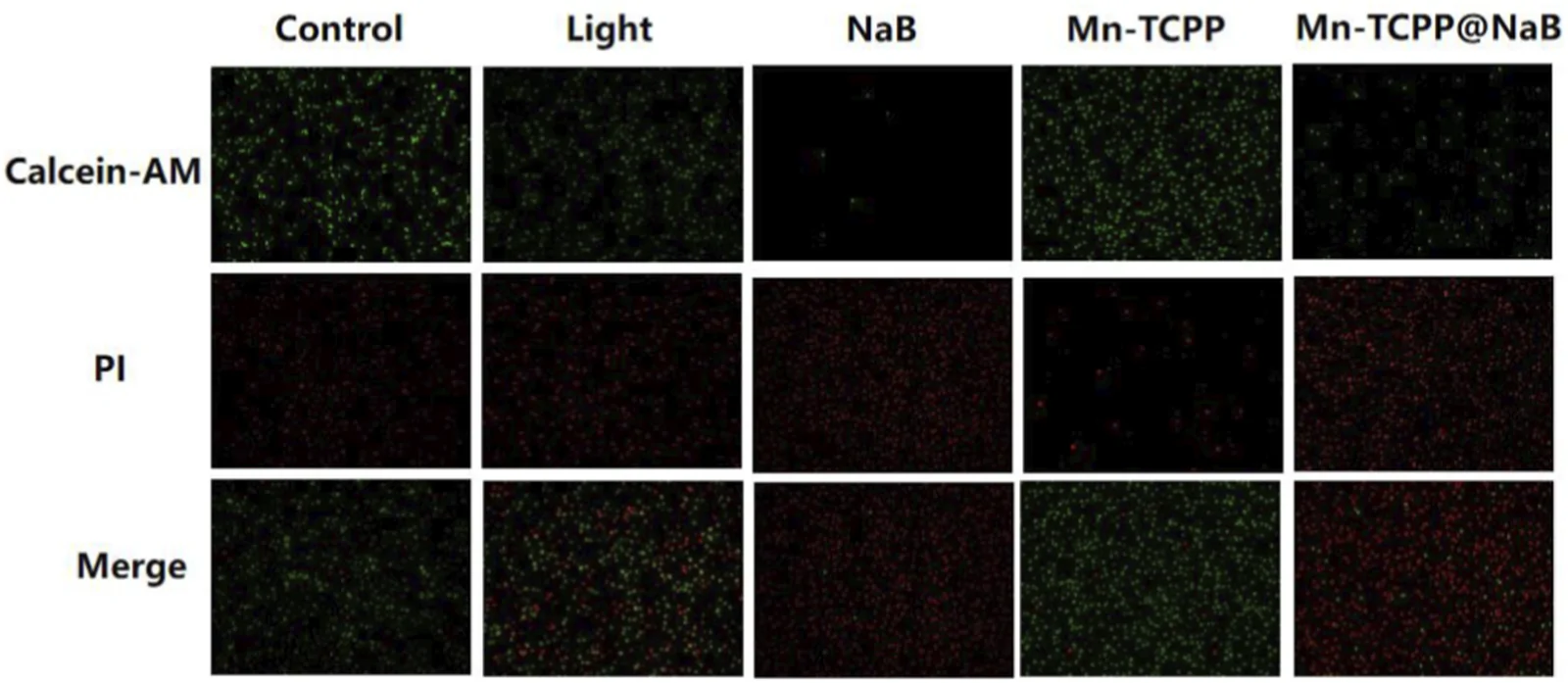
Typical Calcein-AM/propidium iodide of *Escherichia coli* subjected to the following treatments. The fluorescence (green) in Calcein-AM is a sign of relatively intact membrane integrity and propidium iodide is a sign of broken membrane integrity. The control, light, free NaB, Mn-TCPP HOF, and NaB@Mn-TCPP HOF are presented as columns. The images are qualitative.

Figure 7 demonstrates the calcein-AM/PI staining of *E. coli*. Control and Light groups had mainly green fluorescence and little red fluorescence. Free-NaB group exhibited a relative reduction in green signal and enhancement in PI-associated red signal. The Mn-TCPP HOF group that was illuminated maintained high levels of green fluorescence, and the NaB@Mn-TCPP HOF group that was illuminated displayed a red-dominant pattern in the field presented. These representative images can be used with membrane compromise but do not give quantitative statistical data.

**FIGURE 8:**
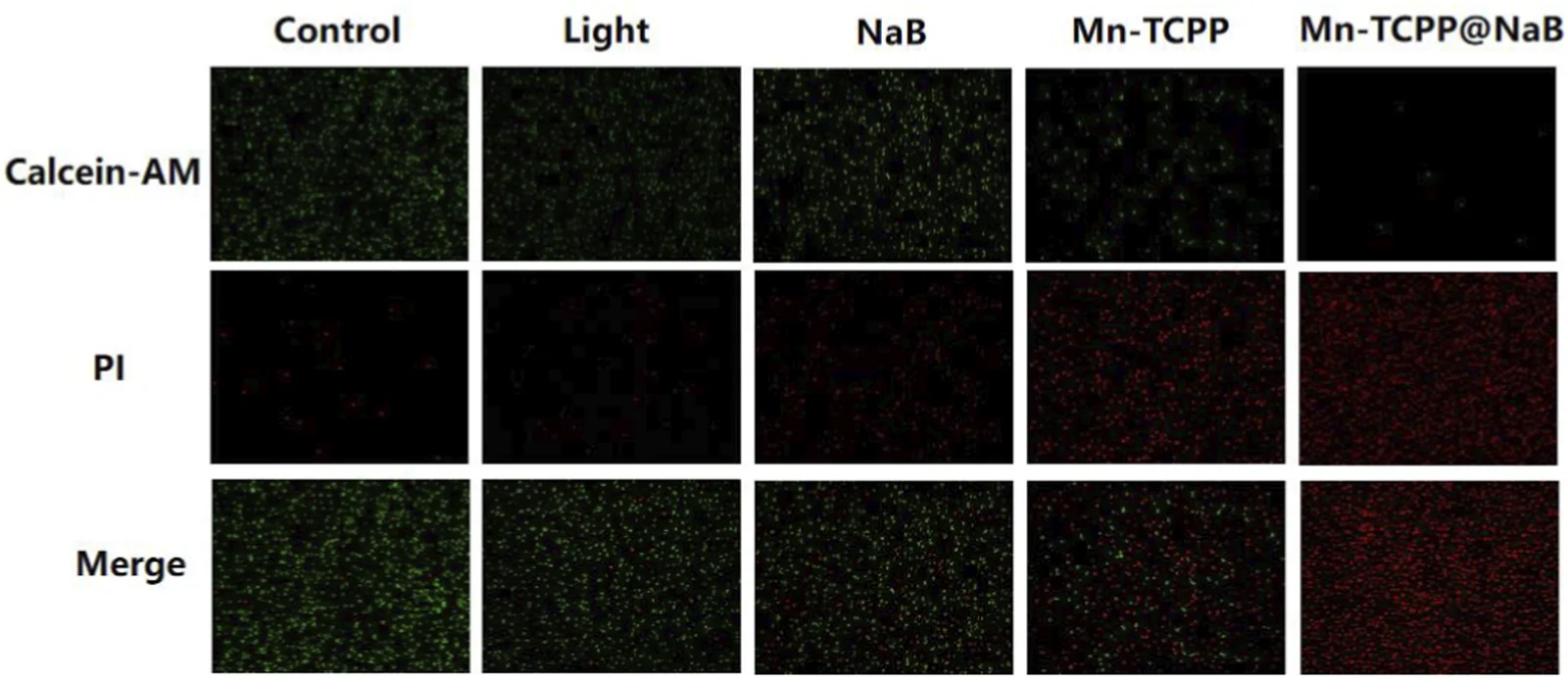
*Staphylococcus aureus* representative Calcein-AM/propidium iodide staining following the treatments mentioned. The fluorescence of calcein-AM (green) is used to determine that the membrane is relatively intact whereas propidium iodide fluorescence (red) is used to determine compromised membrane integrity. Columns indicate Control, Light, free NaB, Mn-TCPP HOF and NaB@Mn-TCPP HOF. Images are interpreted qualitatively.

Calcein-AM/PI staining of *S. aureus* is illustrated in Figure 8. The Control and Light groups were mainly green, the free-NaB group exhibited mixed green and red fluorescence, and the illuminated Mn-TCPP HOF group exhibited apparently decreased green fluorescence with enhanced red signal. The illuminated NaB@Mn-TCPP HOF group also presented a red-dominant pattern in the representative image.

These qualitative observations are consistent with the various levels of membrane damage to the treatment fields presented. They fail to determine the source chemical process, they do not differentiate between the effects of light and dark matter and cannot make any inferential comparison of the fluorescence intensity.

### 3.12 Inhibition of biofilm formation

**FIGURE 9:**
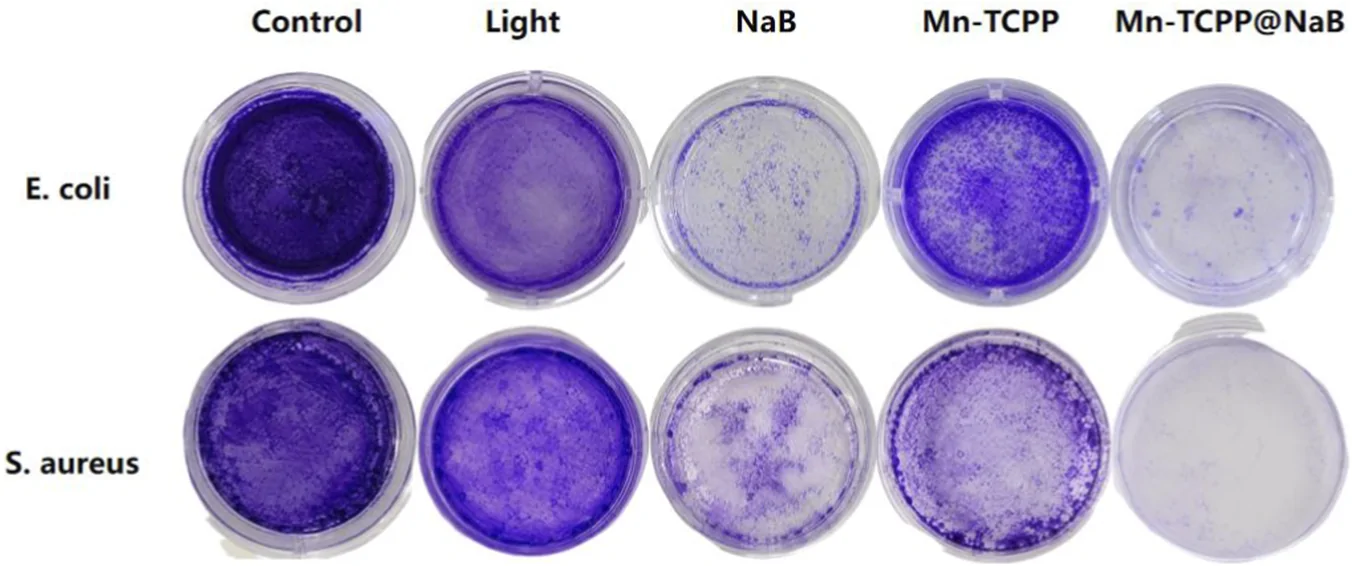
Representative crystal-violet biofilm formation of *Escherichia coli* and *Staphylococcus aureus* after the respective treatments. The two bacterial species are presented in rows and columns present Control, Light, free NaB, Mn-TCPP HOF, and NaB@Mn-TCPP HOF. Purple staining is used to show retained attached biomass. The experiment does not measure eradication of a pre-established mature biofilm but biofilm formation during the 24-h treatment period.

Figure 9 demonstrates crystal-violet staining following the 24-h biofilm-formation period. Both *E. coli* and *S. aureus* Control and Light groups had a significant amount of purple stain. The free-NaB and Mn-TCPP HOF illuminated groups displayed obvious decreases in staining, whereas the NaB@Mn-TCPP HOF wells illuminated displayed less residual staining in the plates shown.

The representative pattern can be used in the inhibition of attached-biomass accumulation during biofilm formation under the tested conditions. Since treatment was done prior to 24-h development time and there is no quantitative absorbance or viable-biofilm data set reported, it cannot be concluded that an established mature biofilm has been eradicated or a statistically resolved difference in biofilm biomass exists.

### 3.13 Bacterial morphology

**FIGURE 10:**
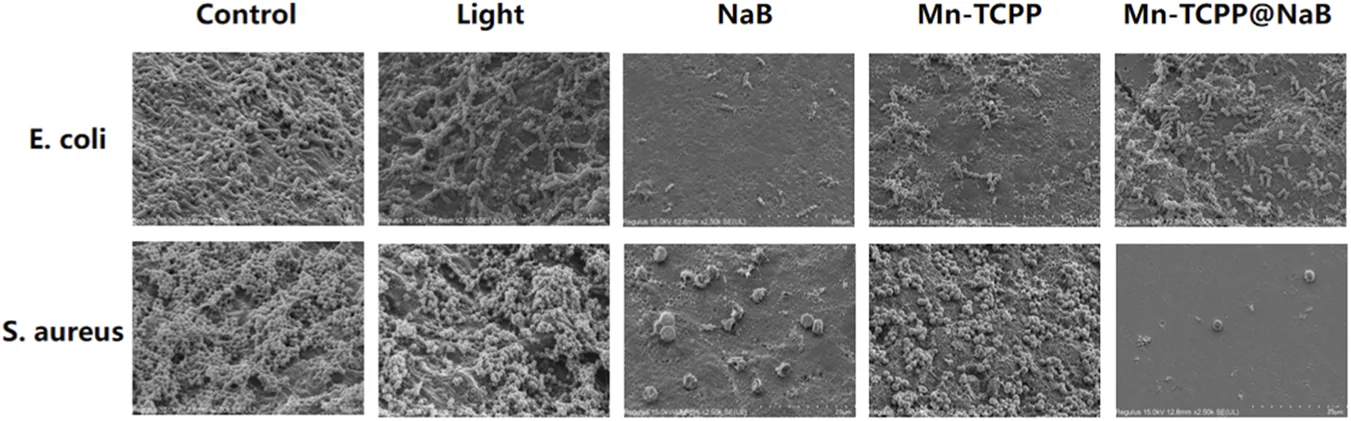
Typical scanning electron microscopy pictures of *Escherichia coli* and *Staphylococcus aureus* following the treatments indicated. Columns indicate Control, Light, free NaB, Mn-TCPP HOF, and NaB@Mn-TCPP HOF. The groups were exposed to 660-nm light at 100 mW cm^−^2 over a period of 10 min: Light, Mn-TCPP HOF, and NaB@Mn-TCPP HOF; the Control and NaB groups were kept in the dark. Qualitative interpretation of images.

Figure 10 presents representative scanning electron microscopy images. *Escherichia coli* in the Control and Light groups were observed as numerous aggregated rods. The free-NaB and Mn-TCPP HOF groups exhibited a visible decrease in surface coverage and aggregation, and the NaB@Mn-TCPP HOF group demonstrated the least apparent surface-associated bacterial coverage among the fields presented.

The Control and Light groups of *S. aureus* were seen to be numerous aggregated cocci. The free-NaB and Mn-TCPP HOF groups exhibited an apparent decrease in the density of bacteria, but there were only sparse cocci or fragment-like structures observed in the representative NaB@Mn-TCPP HOF field.

Due to the fact that magnification and scale presentation were not completely standardized among the available panels, the images are used as a qualitative evidence of the changes in morphology and surface colonization and not used for direct quantitative comparison of bacterial abundance.

### 3.14 Proposed interpretation of antibacterial activity

The preserved porphyrinic absorption of Mn-TCPP HOF allows for consideration of a potential light-related photochemical contribution to the observed antibacterial phenotype. Calcein-AM/PI staining and scanning electron microscopy are consistent with membrane associated injury, but the lack of equivalent dark material controls precludes isolation of a light-specific effect. ROS generation and identity, manganese release, manganese mediated oxidative stress, intracellular targets, and NaB regulated signaling pathways were not directly measured and so are offered as proposed interpretations, not demonstrated mechanisms. The present data are limited to descriptive antibacterial and biofilm formation inhibition observations under the conditions selected.

## 4 Discussion

This paper was a study that prepared NaB@Mn-TCPP HOF and tested its physicochemical properties, apparent loading and release of NaB, hemolytic activity, and antibacterial and biofilm-inhibitory phenotypes on *E. coli* and *S. aureus*. The association of NaB with the parent material is indicated by broad retention of the FTIR and XRD profiles, preservation of the main porphyrinic absorption characteristics, comparable hydrodynamic diameters, colocalization of Na with Mn-TCPP-rich particle areas, and the change in zeta potential. These results agree with the ability of hydrogen-bonded supramolecular substances to entrap guests at mild conditions (Lin et al., 2022; Ya et al., 2024). However, the current description does not specify a particular molecular binding mode or identify pore-confined NaB as opposed to interlayer or surface-associated NaB.

The loading analysis revealed a maximum apparent carrier-mass-normalized value of ca. 56% at a feed ratio of 4 mg NaB: 5 mg carrier. This translates to an estimated NaB content of ca. 36 wt% in the recovered composite and an apparent encapsulation efficiency of ca. 70%. Such definitions are necessary since carrier-relative loading is not identical to the mass fraction of NaB in the final composite. Moreover, UV absorbance at 210 nm offers an indirect estimate susceptible to matrix components and thus should be regarded as apparent loading estimates and not chemically specific absolute measurements.

About 53% of the calculated NaB loaded was released in a period of 12 h. The progressive partial release is supported by the fact that the increase does not reach an end, but it cannot be concluded that the process can be sustained and controlled. The hydrodynamic diameters of the particles were almost the same as before loading and after loading which shows that there was no significant aggregation of the aqueous medium used to test the particle; and zeta potential change implies that the interface between the particle and water has been altered. Both values are related to the dispersion environment and cannot be directly generalized to physiological fluids (Bhattacharjee, 2016).

In the representative biological images, the NaB@Mn-TCPP HOF group was less visible and had a red-dominant PI-staining pattern and decreased coverage of bacterial colonies in the surface-associated fields. The same apparent pattern did not result with light alone. Nevertheless, since there were no corresponding dark Mn-TCPP HOF and dark NaB@Mn-TCPP HOF groups, the effect of illumination cannot be isolated from material-associated dark effects. The findings are consistent with membrane-related injury, however, ROS production was not directly quantified and no particular reactive species can be identified. Qualitative differences between *S. aureus* and *E. coli* still exist and are based on one reference strain of each species.

In the crystal-violet experiment, inhibition of biofilm formation was mainly determined as the treatment was given in 24 h time frame. NaB@Mn-TCPP HOF had the least remaining stains on all the groups shown and the paper did not examine whether a pre-established mature biofilm can be eradicated. The total attached biomass is measured by crystal violet which does not differentiate viable cells, nonviable cells and extracellular matrix material (Peeters et al., 2008). It follows that the current results are termed as inhibition of biofilm development and not the removal of mature-biofilms.

NaB can be part of the composite phenotype as a result of growth or biofilm development. NaB has been reported to have an inhibitory effect on motility, extracellular-polysaccharide synthesis, surface hydrophobicity, quorum-sensing and biofilm gene expression in *Vibrio* parahaemolyticus (Zhu et al., 2022), *Salmonella Typhimurium* biofilm formation (Liu et al., 2022), pathogenic yeasts antimicrobial effects (Nguyen et al., 2011) and ToxT dependent virulence processes in *V. cholerae* (Kundu et al., 2025). It is not possible to suppose that these mechanisms are the same in *E. coli* or *S. aureus* since they were not quantified in the current study. The free-NaB group was also given about 500 μg/mL of NaB, which was lower than the amount provided to the composite group, estimated at 36 μg/mL of NaB, which further makes it impossible to directly compare between-group differences in loading and component interaction.

The manganese contribution is also not solved. Manganese is a necessary bacterial micronutrient, which participates in the functioning of metalloenzymes and oxidative-stress resistance (Juttukonda and Skaar, 2015; Turner et al., 2015), and nanostructures of manganese dioxide can exhibit antibacterial activity by mechanisms that cannot be extrapolated to manganese coordinated in porphyrin HOF (Du et al., 2020). EDS mapping validates Mn in particle regions but does not confirm release, oxidation state or biological activity. Therefore, oxidative stress caused by Mn 2 + and bacterial killing mediated by manganese are considered as hypotheses and not results.

The mean hemolysis values of the material groups were less than 5% at 100 g mL −1 after 2 h. This finding is a preliminary suggestion that there is low erythrocyte lysis in the conditions tested, however, one-concentration assay cannot determine general biocompatibility or host-cell selectivity. Absorbance-based hemolysis measurements can also be affected by nanomaterials (Dobrovolskaia et al., 2008), and further concentration-dependent and light-dependent host-cell evaluation is needed to make wider safety conclusions.

The biological research was a partial factorial study of the material by illumination in five groups and not a full factorial design. The dark material controls were not dose matched to free NaB and composite treatments, nor did they have nonassembled physical-mixture group; therefore, there is no way to distinguish between effects of light, assembly and interactions among components. Microbiological data will be based on images that are representative and do not have an entire quantitative dataset, one ATCC reference strain per species, and ordinary laboratory media. The nominal irradiation rate was 60 J cm^−2^ with no measurements made of irradiance uniformity, spectral bandwidth, or temperature of sample. There are also some other drawbacks such as the lack of direct measurement of ROS and manganese release, formal synergy test, mature-biofilm treatment, staining control of strains, host-cell testing, and physiological media. These results are thus best interpreted as the evidence of proof-of-concept studies, but not as the indication of therapeutic effect or as the solution of multimechanistic path.

## 5 Conclusion

All in all, the main spectroscopic, diffraction and colloidal properties of the parent Mn-TCPP HOF were preserved by NaB@Mn-TCPP HOF following the incorporation of NaB. The apparent load was about 56% at a feed ratio of 4 mg NaB to 5 mg carrier with an estimated value of 36 wt% of NaB content in the recovered composite and an apparent encapsulation rate of approximately 70%. About half (53%) of the expected amount of loaded NaB released itself over 12 h without attaining any final point. At the chosen exploratory parameters, there was a visible decrease in colony growth, attachment of biomass staining and bacterial coverage on the surface as well as PI signal increase in the group of the NaB@Mn-TCPP HOF. Hemolysis was less than 5 percent after 2 hours at 100 μg per mL. It is worth considering additional studies on the light-sensitive platform of NaB@Mn-TCPP HOF. There are still unresolved issues such as specific activity under light, certain ROS pathways, release of manganese, the mechanisms of bacteria that are regulated by NaB, elimination of mature-biofilms, host-cell selectivity and pharmacological interactions in terms of quantity.

## Data Availability

The raw data supporting the conclusions of this article will be made available by the authors, without undue reservation.

## References

[B1] Antimicrobial Resistance (2022). Global burden of bacterial antimicrobial resistance in 2019: a systematic analysis. Lancet 399 (10325), 629–655. 10.1016/S0140-6736(21)02724-0 35065702 PMC8841637

[B2] Beirao S. Fernandes S. Coelho J. Faustino M. A. F. Tomé J. P. C. Neves M. G. P. M. S. (2014). Photodynamic inactivation of bacterial and yeast biofilms with a cationic porphyrin. Photochem Photobiol. 90 (6), 1387–1396. 10.1111/php.12331 25112506

[B3] Bhattacharjee S. (2016). DLS and zeta potential—What they are and what they are not? J. Control Release 235, 337–351. 10.1016/j.jconrel.2016.06.017 27297779

[B4] Chen F. F. L.S. Zhang L.Y. Wang H.Y. Zhu K. Ma N.N. Yang (2026). Microneedle-mediated delivery of an NIR-triggered nanoagent for synergistic anti-biofilm infection therapy. Eur. Cell Mater 55, 63–79. 10.22203/ecm.v055a05

[B5] Chou T. C. (2010). Drug combination studies and their synergy quantification using the Chou–Talalay method. Cancer Res. 70, 440–446. 10.1158/0008-5472.CAN-09-1947 20068163

[B6] Dobrovolskaia M. A. Clogston J. D. Neun B. W. Hall J. B. Patri A. K. McNeil S. E. (2008). Method for analysis of nanoparticle hemolytic properties *in vitro* . Nano Lett. 8 (8), 2180–2187. 10.1021/nl0805615 18605701 PMC2613576

[B7] Du T. Chen S. Zhang J. Li T. Li P. Liu J. (2020). Antibacterial activity of manganese dioxide nanosheets by ROS-Mediated pathways and destroying membrane integrity. Nanomater. (Basel) 10 (8), 1545. 10.3390/nano10081545 32784527 PMC7466589

[B8] Flemming H. C. Wingender J. Szewzyk U. Steinberg P. Rice S. A. Kjelleberg S. (2016). Biofilms: an emergent form of bacterial life. Nat. Rev. Microbiol. 14 (9), 563–575. 10.1038/nrmicro.2016.94 27510863

[B9] Foucquier J. Guedj M. (2015). Analysis of drug combinations: current methodological landscape. Pharmacol. Res. Perspect. 3 (3), e00149. 10.1002/prp2.149 26171228 PMC4492765

[B10] Grodzicka M. G. Gąsior T. Jędrzejewski S. Wrotek P. Madajski A. Radtke (2025). Porous iron scaffolds with the addition of manganese and copper—physicochemical characteristics and biocompatibility analysis. Eur. Cell Mater 54, 128–144. 10.22203/ecm.v054a09

[B11] He X. T. Luo Y. H. Zheng Z. Y. Wang C. Wang J. Y. Hong D. L. (2019). Porphyrin-Based hydrogen-bonded organic frameworks for the photocatalytic degradation of 9,10-Diphenylanthracene. ACS Appl. Nano Mater. 2 (12), 7719–7727. 10.1021/acsanm.9b01787

[B12] Juttukonda L. J. Skaar E. P. (2015). Manganese homeostasis and utilization in pathogenic bacteria. Mol. Microbiol. 97 (2), 216–228. 10.1111/mmi.13034 25898914 PMC4631260

[B13] Kashef N. Hamblin M. R. (2017). Can microbial cells develop resistance to oxidative stress in antimicrobial photodynamic inactivation? Drug Resist Updat 31, 31–42. 10.1016/j.drup.2017.07.003 28867242 PMC5673603

[B14] Kundu S. Das S. Maitra P. Halder P. Koley H. Mukhopadhyay A. K. (2025). Sodium butyrate inhibits the expression of virulence factors in *Vibrio cholerae* by targeting ToxT protein. mSphere 10 (5), e0082424. 10.1128/msphere.00824-24 40261078 PMC12108080

[B15] Lin Z. J. Mahammed S. A. R. Liu T. F. Cao R. (2022). Multifunctional porous hydrogen-bonded organic frameworks: current status and future perspectives. ACS Cent. Sci. 8 (12), 1589–1608. 10.1021/acscentsci.2c01196 36589879 PMC9801510

[B16] Liu J. Zhu W. Qin N. Ren X. Xia X. (2022). Propionate and butyrate inhibit biofilm Formation of Salmonella Typhimurium grown in laboratory media and food models. Foods 11 (21), 3493. 10.3390/foods11213493 36360105 PMC9654251

[B17] Maisch T. (2015). Resistance in antimicrobial photodynamic inactivation of bacteria. Photochem Photobiol. Sci. 14 (8), 1518–1526. 10.1039/c5pp00037h 26098395

[B18] Nguyen L. N. Lopes L. C. L. Cordero R. J. B. Nosanchuk J. D. (2011). Sodium butyrate inhibits pathogenic yeast growth and enhances the functions of macrophages. J. Antimicrob. Chemother. 66 (11), 2573–2580. 10.1093/jac/dkr358 21911344

[B19] Peeters E. Nelis H. J. Coenye T. (2008). Comparison of multiple methods for quantification of microbial biofilms grown in microtiter plates. J. Microbiol. Methods 72, 157–165. 10.1016/j.mimet.2007.11.010 18155789

[B20] Pereira M. A. Faustino M. A. F. Tomé J. P. C. Neves M. G. P. M. S. Tomé A. C. Cavaleiro J. A. S. (2014). Influence of external bacterial structures on the efficiency of photodynamic inactivation by a cationic porphyrin. Photochem Photobiol. Sci. 13 (4), 680–690. 10.1039/c3pp50408e 24549049

[B21] Sabino C. P. Ribeiro M. S. Wainwright M. Dos Anjos C. Sellera F. P. Dropa M. (2023). The biochemical mechanisms of antimicrobial photodynamic Therapy(†). Photochem Photobiol. 99 (2), 742–750. 10.1111/php.13685 35913428

[B22] Stewart P. S. Costerton J. W. (2001). Antibiotic resistance of bacteria in biofilms. Lancet 358 (9276), 135–138. 10.1016/s0140-6736(01)05321-1 11463434

[B23] Turner A. G. Ong C. L. Y. Gillen C. M. Davies M. R. West N. P. McEwan A. G. (2015). Manganese homeostasis in group A Streptococcus is critical for resistance to oxidative stress and virulence. mBio 6 (2), e00278. 10.1128/mBio.00278-15 25805729 PMC4453566

[B24] Wainwright M. Maisch T. Nonell S. Plaetzer K. Almeida A. Tegos G. P. (2017). Photoantimicrobials-are we afraid of the light? Lancet Infect. Dis. 17 (2), e49–e55. 10.1016/S1473-3099(16)30268-7 27884621 PMC5280084

[B25] Xu Q. Zhan G. Zhang Z. Yong T. Yang X. Gan L. (2021). Manganese porphyrin-based metal-organic framework for synergistic sonodynamic therapy and ferroptosis in hypoxic tumors. Theranostics 11 (4), 1937–1952. 10.7150/thno.45511 33408790 PMC7778611

[B26] Ya J. Zhang H. Qin G. Huang C. Zhao C. Ren J. (2024). A biocompatible Hydrogen-Bonded Organic Framework (HOF) as sonosensitizer and artificial enzyme for In-Depth treatment of Alzheimer's Disease. Adv. Healthc. Mater 13 (29), e2402342. 10.1002/adhm.202402342 39031538

[B27] Zhu W. X. Gao J. Liu H. Liu J. Jin T. Qin N. (2022). Anti-biofilm effect of sodium butyrate against Vibrio parahaemolyticus. Food control., 131 (27), 108422. 10.1016/j.foodcont.2021.108422

